# Long-term Safety, Efficacy, and PROs: Phase 3 Study of Leuprolide Acetate 6-month IM Depot in Central Precocious Puberty

**DOI:** 10.1210/jendso/bvaf224

**Published:** 2026-01-30

**Authors:** Karen O Klein, Nelly Mauras, Sunil Nayak, Bhuvana Sunil, Ahmed M Soliman, Alvina R Kansra

**Affiliations:** Department of Pediatrics, University of California San Diego School of Medicine and Rady Children's Hospital, San Diego, CA 92123, USA; Nemours Children's Health, Jacksonville, FL 32207, USA; Pediatric Endocrine Associates, Greenwood Village, CO 80111, USA; University of Colorado School of Medicine, Aurora, CO 80045, USA; Mary Bridge Children's Hospital, Tacoma, WA 98403, USA; AbbVie Inc., North Chicago, IL 60064, USA; AbbVie Inc., North Chicago, IL 60064, USA

**Keywords:** central precocious puberty, gonadotropin-releasing hormone agonist, intramuscular, leuprolide acetate, long-term treatment, children

## Abstract

**Context:**

The long-term safety and efficacy of leuprolide acetate (LA) intramuscular (IM) depot in children with central precocious puberty (CPP) have not been examined.

**Objective:**

We evaluated the 144-week efficacy and safety of LA IM depot with 6-month dosing frequency for the treatment of CPP.

**Methods:**

Children with CPP received 45 mg LA IM depot in a phase 3, single-arm study (NCT03695237) that enrolled both treatment-naive (n = 27) and previously treated (n = 18) children (age: 7.8 ± 1.27 years). Outcomes included suppression of peak-stimulated LH (<4 mIU/mL), basal estradiol (<20 pg/mL), and testosterone (<30 ng/dL) and physical puberty signs (based on nonmissing data), height-related outcomes, patient/parent-reported outcomes, and safety.

**Results:**

Peak-stimulated LH was suppressed in 93%, 95%, 100%, and 100% of children at weeks 72, 96, 120, and 144, respectively; basal sex hormones were suppressed in all children. Most children had pubertal signs suppressed during long-term treatment (Tanner staging, weeks 72-144, girls: 86.4-94.9%; boys, 50.0-75.0%). After week 48, mean incremental height velocity remained relatively stable, and mean ratio of bone age to chronological age was 1.2. At week 144, mean annualized change from baseline in predicted adult height (PAH) was 1.4 cm/year, and mean absolute PAH in girls improved compared with mid-parental height. Health-related quality of life was maintained. No new related safety concerns were identified.

**Conclusion:**

Six-month LA IM depot for 144 weeks demonstrated a sustained inhibition of the GnRH axis in children with CPP with an acceptable safety profile. Health-related quality of life was maintained, and PAH improved during ∼3 years of treatment.

Treatment of children with central precocious puberty (CPP) includes suppression of the gonadotropin releasing hormone (GnRH) axis to halt further progression of the physical changes of puberty and to preserve adult height potential. For more than 2 decades, the standard of care for CPP has included GnRH analogs (GnRHa). Over the years, several long-term dosing options have become available, which have significantly decreased the frequency of injections for children with CPP. Six-month dosing formulations have been shown to have comparable safety and efficacy over 48 weeks of treatment compared with those previously observed with shorter dosing intervals [1-5]. However, long-term safety and efficacy have not been examined for leuprolide acetate (LA) 45 mg 6-month formulations thus far [1, 2].

Long-term follow-up is important to help assess the maintenance of pubertal suppression and to more comprehensively examine height-related outcomes, including predicted adult height (PAH). It is also important to assess patient/parent-reported outcomes (PROs) because these data are generally lacking for GnRHa treatment in children with CPP. Of the few studies that have been conducted, some have shown that GnRHa treatment may be associated with a decline in the physical functioning component of health-related quality of life [6, 7]. However, additional data are needed to confirm these findings, because 1 of these studies was a cross-sectional assessment of children with CPP vs controls matched for age [7] and the other prospective study combined children with CPP and those with early-fast puberty [6] in their analysis.

Here, we report the long-term safety, efficacy, and PROs examined over 144 weeks of treatment with LA 45 mg 6-month intramuscular (IM) depot among children with CPP in a phase 3, multicenter, open-label study, expanding on results from the initial 48-week follow-up reported previously [2].

## Materials and Methods

### Population

Participants included both GnRHa-naive and previously treated children diagnosed with CPP based on the appearance of pubertal changes before chronological age (CA) of 8 years among girls or 9 years among boys and bone age (BA) being advanced ≥1 year over CA. All children were initially determined to have premature activation of the hypothalamic-pituitary-gonadal axis as the cause of their CPP and were excluded if they had a diagnosis of short stature (height more than 2.25 SD below the mean for age and sex); peripheral precocious puberty; or any other pituitary, hypothalamic, adrenal, thyroid, or gonadal function abnormalities. Children who were GnRHa-naive had to have a peak-stimulated luteinizing hormone (LH) of ≥6 mIU/mL at screening. Girls who were GnRHa-naive needed to be 2 to 8 years old and have breast pubertal Tanner stage ≥2 and BA <13 years, while boys who were GnRHa-naive had to be 2 to 9 years old and have testicular volume ≥4 cm^3^ and BA <14 years. Children who were previously treated needed to be treated with any GnRHa therapy for ≥6 months prior to enrollment, have documented maintenance of peak stimulated LH <4 mIU/mL at screening, and have a CA of 2 to 10 years among girls and 2 to 11 years among boys.

### Study Design

This study was a phase 3, single-arm, open-label, multicenter (16 sites) study examining up to 144 weeks of treatment (up to 6 total doses) with LA 45-mg 6-month IM depot [8] over 2 parts. Part 1 was reported previously [2] and included an initial 48 weeks of treatment (dose 1 at baseline and dose 2 at week 24) and 2 postbaseline study visits (week 24 and 48). Immediately after completing the assessments in part 1, children continued to a long-term treatment extension and received their third dose at week 48. In part 2, study visits were conducted at week 72 and every 24 weeks until the participant completed all planned doses (last planned dose was at week 120) or earlier if the participant prematurely discontinued study treatment. Overall long-term efficacy, including maintenance of LH suppression, safety, and PROs, are presented here (first patient visit, October 24, 2018; last patient visit, November 29, 2023).

The study was conducted in accordance with the International Council for Harmonisation of Technical Requirements for Pharmaceuticals for Human Use protocol, applicable regulations, and guidelines governing clinical study conduct and ethical principles that have their origin in the Declaration of Helsinki. Institutional review board approval was obtained at each participating site, written informed consent was provided by the parents or legal guardians, and child assent was obtained as appropriate.

### Study Intervention

LA 45 mg 6-month IM depot (LUPRON DEPOT, AbbVie Inc., North Chicago, IL) [8] was administered as an IM injection once every 24 weeks as previously described [2]. The 6-month formulation used in this study is the same as that approved for prostate cancer and is delivered in the same 1.5-mL injection volume as the 3-month LA IM depot approved for CPP [8, 9]. Duration of treatment was assessed as the last dose date minus the first dose date plus 1 day. Noncompliance with the study dosing scheme was evaluated based on a gap of >3 days between the last day of injection and the day of the next injection.

### Long-term Efficacy Assessments

Efficacy outcomes were assessed at baseline and at week 24, 48, 72, 96, 120, and 144 study visits, unless otherwise specified.

#### Hormone measurements

Basal LH, follicle-stimulating hormone (FSH), and sex hormones (testosterone and estradiol) were obtained before carrying out stimulation tests to measure peak-stimulated LH and FSH using 20-μg/kg generic aqueous LA subcutaneous injection. Peak-stimulated LH and FSH concentrations were recorded at 30 and 60 minutes. A lower limit of quantification of 1 mIU/mL and range of 1 to 100 mIU/mL were used for an ELISA of LH and FSH levels at a central laboratory using commercially available assays (LH, Alpco Diagnostics Catalog# 11-LUTHU-E01, RRID: AB_2936342 and Diagnostics Biochem Canada Catalog# CAN-LH-4040, RRID: AB_2936341; FSH, Enzo Life Sciences Catalog# ENZ-KIT108, RRID: AB_2909630). Sex hormone levels were measured using liquid chromatography–mass spectrometry (Bioanalysis, AbbVie Inc.), with a lower limit of quantification of 0.025 ng/dL for testosterone and 3 pg/mL for estradiol.

#### Physical signs of puberty

The description of physical signs provided here focus on part 2 as the description for part 1 was previously published [2]. Clinical suppression of physical signs of puberty was assessed using a modified Tanner pubertal staging and defined as regression or no progression of palpable breast development among girls or testicular volume and genital staging among boys. The presence of menstrual bleeding, changes in uterine length/volume and endometrial stripe presence (assessed by ultrasound in girls), and changes in testicular volume (assessed by an examiner in boys) were also assessed and documented at each study visit.

#### Height- and weight-related assessments

Height was measured in triplicate using the same standard stadiometer equipment in each participant during the trial, including a Harpenden stadiometer or recumbent length table. Height velocity (cm/year) was calculated by using 2 measurements separated by ≥6 months; for the baseline measurement, a historic height obtained ≥6 months prior to screening was used. The BA/CA ratio was obtained for each child at the time of the BA measurement. Readings of BA radiographs of the left hand and wrist were performed at a facility specified by the study investigator and reviewed by a central reader using the FELS BA measurement [10, 11] obtained from the BoneXpert automated system (Visiana ApS, Hørsholm, Denmark) [12]. PAH was estimated using the Bayley-Pinneau method [13 ] by dividing actual height by the average percentage of adult height associated with the concurrent BA. A unique BA was matched with a single height measurement that was collected within ≤30 days before and ≤90 days after the BA assessment. PAH values were calculated between study visits and expressed as annualized change from baseline in cm/year.

Weight was assessed during screening, at baseline, and during study visits and used to calculate the body mass index (BMI) for all children.

### PRO Assessments

The participant's parent or legal guardian completed both PRO instruments used in this study. The Pediatric Quality of Life Inventory™ (PedsQL) Parent-Proxy Report (23 questions in total) [14, 15] was administered to parents at weeks 24, 72, and 144. Assessments were performed separately for 3 different age groups (toddler, 2-4 years; young child, 5-7 years; child, 8-12 years). The PedsQL total scale scores range from 0 to 100, with higher scores indicating a better health-related quality of life. Scores for the 4 underlying domains of PedsQL (physical functioning, emotional functioning, social functioning, and school functioning) were also reported. The Patient-Reported Outcomes Measurement Information System® (PROMIS) Peer Relationships Guardian Proxy (short form, 7 questions in total) [16, 17] was administered to parents/guardians of all children aged ≥5 years at every study visit. The mean PROMIS Peer Relationships T-score for the US general population is 50; higher scores reflect better peer relationships.

### Long-term Safety Assessments

Evaluations were performed from baseline through week 144 and included assessments of adverse events (AEs) and AEs of special interest (AESI), physical/vital signs, and clinical laboratory values. AEs were collected and coded using the Medical Dictionary for Regulatory Activities Version 24.1 (MedDRA). AESI were retrieved by standard MedDRA query or company MedDRA query searches to identify AEs potentially related to the following: biochemical acute-on-chronic response, bone fracture, convulsion/seizure, epiphysiolysis, hormonal flare response and related psychiatric/mood event, hypersensitivity reaction, injection site reaction (ISR), and neuropsychiatric event. In addition to standard AE monitoring, ISRs and hormonal flare responses were also recorded through patient/parent questionnaires administered by study staff. ISRs were evaluated during study drug administration as well as during peak-stimulated hormone testing, and a questionnaire administered after each injection included questions regarding whether the following symptoms were observed: drainage or abscess, the presence of injection site pain or tenderness, redness or swelling, and skin warmth. Per the protocol, it was mandated to report all ISRs as AEs. Using a company search of various MedDRA preferred terms (eg, abdominal pain, bone pain, or constipation), AEs potentially related to hormonal flare were evaluated ≤14 days after each study drug dose.

### Statistical Methods

No formal sample size calculation was performed, and 40 patients were considered to be sufficient to support the efficacy and safety analysis for this therapeutic class and patient population (based on previous studies and precedents used for registration), providing an observed response rate of suppression of peak-stimulated LH that is within 16.7% of the true response rate with 95% confidence.

All data were summarized descriptively without formal statistical comparisons. Missing data were not imputed. In the responder analysis in part 2, children with missing data points owing to missed measurement or premature study discontinuation were excluded from the analyses (and were not considered as nonresponders) at the time point being analyzed. Thresholds for responder analyses of hormonal suppression were specified a priori (peak-stimulated LH, <4 mIU/mL; basal estradiol, <20 pg/mL; basal testosterone, <30 ng/dL) as well as post hoc (basal estradiol, <10 pg/mL). The categorical efficacy endpoints were summarized with 95% confidence interval (CI) based on the binomial distribution (Clopper-Pearson exact method). The continuous secondary efficacy endpoints were summarized by sample size, mean, median, SD, SEM, minimum, and maximum, as appropriate. Statistical analyses were performed with SAS software package version 9.4 or later (SAS Institute Inc., Cary, NC) under the UNIX operating system.

## Results

### Patient Population and Study Treatment Exposures

**Figure 1.**
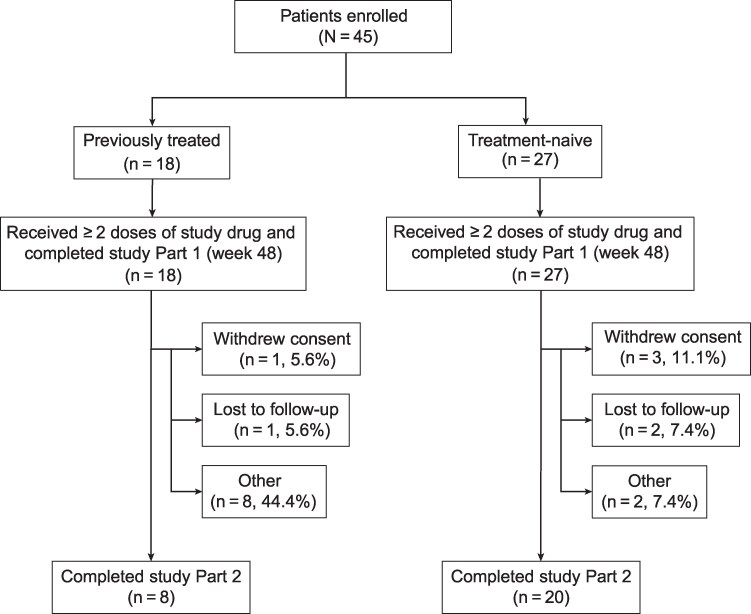
Flow diagram of study participants. Children who discontinued from the study were counted under each reason given for discontinuation; therefore, the sum of reasons for discontinuation may be greater than the overall number of discontinuations. “Other” reasons for discontinuation included: provider, caregiver, or child was no longer interested in treatment and wanted the child to restart puberty.

Of the 45 enrolled children, 28 completed part 2 (Fig. 1). Baseline characteristics are reported in Table 1. Among previously treated children, the median duration of LA treatment was 925 days (min, 337; max, 1029), the median number of injections was 6 (min, 2; max, 6), and 33.3% had a ≤3-day gap between scheduled doses. Among treatment-naive children, the median duration of treatment and number of injections were 1008 days (min, 502; max, 1028) and 6 injections (min, 3; max, 6), respectively, with 48.1% having a ≤3-day gap between scheduled doses.

**Table 1.** Baseline characteristics in children with CPP treated with LA 45-mg 6-month IM depot

Characteristic*^a^*	Previously treated*^b^*	Treatment-naive	Overall
Girls and boys, n	18	27	45
Age, years	8.1 ± 1.73 (4.0, 10.0)	7.7 ± 0.83 (5.0, 9.0)	7.8 ± 1.27 (4.0, 10.0)
BMI standardized score	1.1 ± 1.04 (−1.5, 2.0)	0.8 ± 1.12 (−2.0, 2.0)	0.9 ± 1.08 (−2.0, 2.0)
Height standardized score	1.4 ± 0.97 (−1.0, 2.0)	1.4 ± 0.74 (−0.5, 2.0)	1.4 ± 0.83 (−1.0, 2.0)
Girls, n	17	24	41
Tanner breast stage			
1	4 (23.5)	0	4 (9.8)
2	4 (23.5)	1 (4.2)	5 (12.2)
3	6 (35.3)	18 (75.0)	24 (58.5)
4	3 (17.6)	5 (20.8)	8 (19.5)
Time from puberty onset to treatment, years	1.1 ± 0.93 (0.1, 2.7)	1.4 ± 0.94 (0.2, 5.3)	1.3 ± 0.93 (0.1, 5.3)
Height velocity, cm/year	5.8 ± 2.44 (0.9, 9.4)	10.0 ± 3.27 (5.3, 19.8)	8.3 ± 3.61 (0.9, 19.8)
BA, years	10.4 ± 1.80 (6.9, 12.6)	10.9 ± 0.76 (9.4, 13.0)	10.7 ± 1.30 (6.9, 13.0)
BA/CA	1.3 ± 0.18 (0.9, 1.6)	1.4 ± 0.13 (1.2, 1.8)	1.3 ± 0.16 (0.9, 1.8)
BA−CA, years	1.9 ± 1.01 (−0.7, 3.8)	2.8 ± 0.82 (1.5, 4.7)	2.5 ± 1.00 (−0.7, 4.7)
Boys, n	1	3	4
Testicular volume, cm^3^	3.0	8.0 ± 2.00 (6.0, 10.0)	6.8 ± 2.99 (3.0, 10.0)
Time from puberty onset to treatment, years	6.0	2.4 ± 1.52 (1.2, 4.1)	3.3 ± 2.22 (1.2, 6.0)
Height velocity, cm/year	6.2	10.8 ± 1.51 (9.1, 11.9)	9.7 ± 2.65 (6.2, 11.9)
BA, years	11.5	12.6 ± 1.28 (11.2 13.7)	12.3 ± 1.17 (11.2, 13.7)
BA/CA	1.1	1.4 ± 0.07 (1.3, 1.4)	1.3 ± 0.13 (1.1, 1.4)
BA−CA, years	1.4	3.4 ± 0.48 (3.0, 3.9)	2.9 ± 1.07 (1.4, 3.9)

Abbreviations: BA, bone age; BMI, body mass index; CA, chronological age; CPP, central precocious puberty; IM, intramuscular; LA, leuprolide acetate.

^a^Data are expressed as mean ± SD (range) or number (%), unless otherwise stated.

^b^All previously treated children received prior GnRH analog therapy for ≥6 months prior to enrollment (n = 15, leuprorelin; n = 3, triptorelin; n = 3, histrelin).

### LH and FSH Suppression

**Figure 2.**
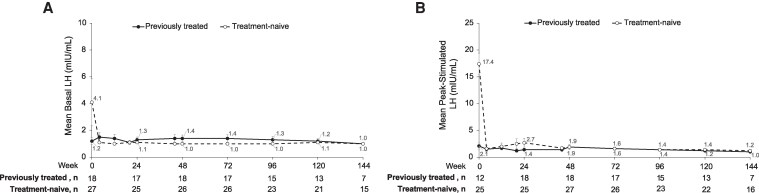
Mean (A) plasma basal and (B) peak-stimulated LH levels in previously treated (solid) and treatment-naive (dotted) children. Week 0 indicates baseline assessment prior to first dose (ie, day 1 of treatment). Vertical lines represent SE bars. In some cases, SE values were too small to visualize. Abbreviation: LH, luteinizing hormone.

**Figure 3.**
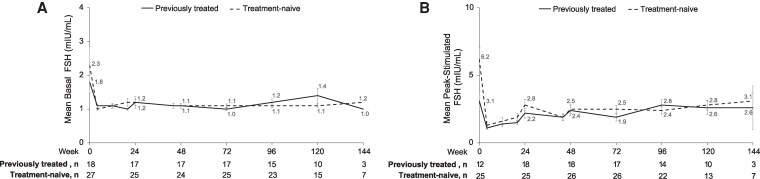
Mean (A) plasma basal and (B) peak-stimulated FSH levels in previously treated (solid) and treatment-naive (dotted) children. Week 0 indicates baseline assessment prior to first dose (ie, day 1 of treatment). Vertical lines represent SE bars. Abbreviation: Follicle-stimulating hormone.

The mean basal (Fig. 2A) and peak-stimulated (Fig. 2B) LH concentrations remained relatively stable and at expected levels for GnRHa treatment through week 144 in both patient groups after immediately declining in treatment-naive children after starting LA. The mean basal and peak-stimulated FSH concentrations were consistent with the observed LH profile (Fig. 3A and 3B).

Peak-stimulated LH was suppressed to <4 mIU/mL in 86.7% (39/45) and 88.9% (40/45) children at week 24 and 48, respectively (analysis was based on all enrolled children). A description of children who were not suppressed by the initial 48 weeks of treatment was detailed previously [2]. After the third dose, 93% (40/43), 95% (36/38), 100% (35/35), and 100% (23/23) of children were suppressed to <4 mIU/mL at weeks 72, 96, 120, and 144, respectively (analysis was based on nonmissing data; ie, children with a missing measurement or premature study discontinuation were excluded from the analysis). Of the 3 children who were not suppressed at week 72, 2 (1 girl and 1 boy) remained unsuppressed even at week 96 (Table 2).

**Table 2.** Patients failing LH suppression at week 72 and 96

Sex (age, years)	Treatment status	Visit/week	LH peak, mIU/mL	Basal E2, pg/mL	Basal T, ng/dL
Female (8)	Previously treated	Baseline	7.73	<3.00*^b^*	NA
		24	4.35	<3.00*^b^*	NA
		48	4.39	<3.00*^b^*	NA
		72	4.84	<3.00*^b^*	NA
		96	3.59	<3.00*^b^*	NA
		120	Missing	Missing	NA
		144	NA*^a^*	NA*^a^*	NA
Female (9)	Previously treated	Baseline	1.20	<3.00*^b^*	NA
		24	3.67	3.22	NA
		48	5.67	Missing	NA
		72	4.05	<3.00*^b^*	NA
		96	4.29	<3.00*^b^*	NA
		120	2.94	7.40	NA
		144	NA*^a^*	NA*^a^*	NA
Male (9)	Treatment-naive	Baseline	16.98	NA	418.0
		24	5.30	NA	7.00
		48	4.51	NA	10.40
		72	5.46	NA	11.70
		96	4.61	NA	10.10
		120	3.21	NA	6.44
		144	Missing	NA	Missing

Abbreviations: E2, estradiol; LH, leuteinizing hormone; NA, not applicable; T, testosterone.

^a^Last injection was administered at week 96.

^b^Lower limit of quantitation.

### Girls: Suppression of Basal Estradiol and Physical Characteristics of Puberty

**Figure 4.**
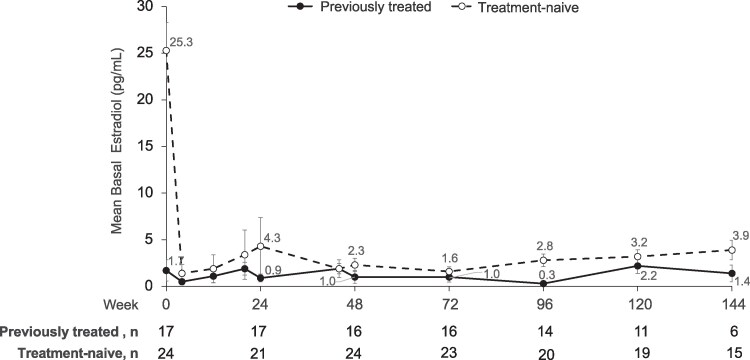
Mean plasma basal estradiol levels in previously treated (solid) and treatment-naive (dotted) girls. Week 0 indicates baseline assessment prior to first dose (ie, day 1 of treatment). Vertical lines represent SE bars.

The mean basal estradiol levels remained relatively stable and at expected GnRHa treatment levels through week 144 in both patient groups after immediately declining in treatment-naive girls after starting LA (Fig. 4). Basal estradiol was suppressed to <20 pg/mL in 97.4% of girls at week 24% and 100% at weeks 48, 72, 96, 120, and 144. Using a more stringent threshold of <10 pg/mL in a post hoc review of data from week 72 to 144, all girls had estradiol values <10 ng/mL at all study visits except 1 treatment-naive girl (baseline age, 8) who had basal estradiol of 15.3 pg/mL at week 144. Her baseline level was 16.2 pg/mL, and all other postbaseline measurements were <10 pg/mL.

After the third study dose, none of the girls experienced menstrual bleeding, and most girls had physical signs of puberty suppressed based on Tanner staging (previously treated, >80%; treatment-naive, >85%). Mean uterine length and volume remained relatively stable or decreased through week 144 (mean [SD] change from baseline to week 144: 0.2 [0.58] cm and −1.0 [1.66] mL in previously treated and −1.2 [1.74] cm and −9.8 [11.80] mL in treatment-naive girls, respectively). At week 144, endometrial stripe was present in 2/4 of previously treated girls and 1/3 of treatment-naive girls who did not have it present at baseline. Endometrial stripe persisted from baseline through week 144 in 1/1 previously treated girl and 8/13 treatment-naive girls.

### Boys: Suppression of Basal Testosterone and Physical Characteristics of Puberty

**Figure 5.**
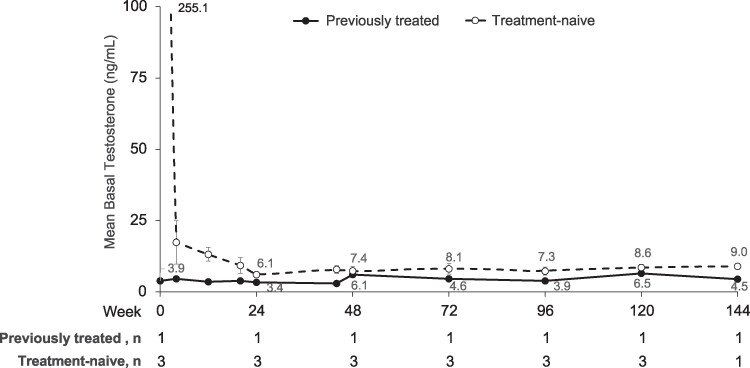
Mean plasma basal testosterone levels in previously treated (solid) and treatment-naive (dotted) boys. Week 0 indicates baseline assessment prior to first dose (ie, day 1 of treatment). Vertical lines represent SE bars. Because n = 1 for the previously treated population, no SE values were available.

All boys had basal testosterone suppressed to <30 ng/dL at all postbaseline study visits through week 144 (Fig. 5; analysis of part 2 was based on nonmissing data). The single enrolled previously treated boy continued to maintain testosterone suppression (≤6.5 ng/dL) postbaseline through week 144. After the third dose, his pubertal stage increased from Tanner 2 at week 72 to Tanner 3 at week 144. His testicular volume increased by 2.0 mL at week 144.

In treatment-naive boys, mean basal testosterone levels declined from 255.1 ng/dL at baseline and remained <10 ng/dL through week 144. After their third dose, 2 of 3 treatment-naive boys examined at weeks 72, 96, and 120 and a single boy examined at week 144 had physical signs of puberty suppressed. Their mean testicular volume declined from baseline by −0.7 mL at week 72 (n = 2) and by −4.0 mL at week 144 (n = 1).

### Height and Weight Outcomes

**Figure 6.**
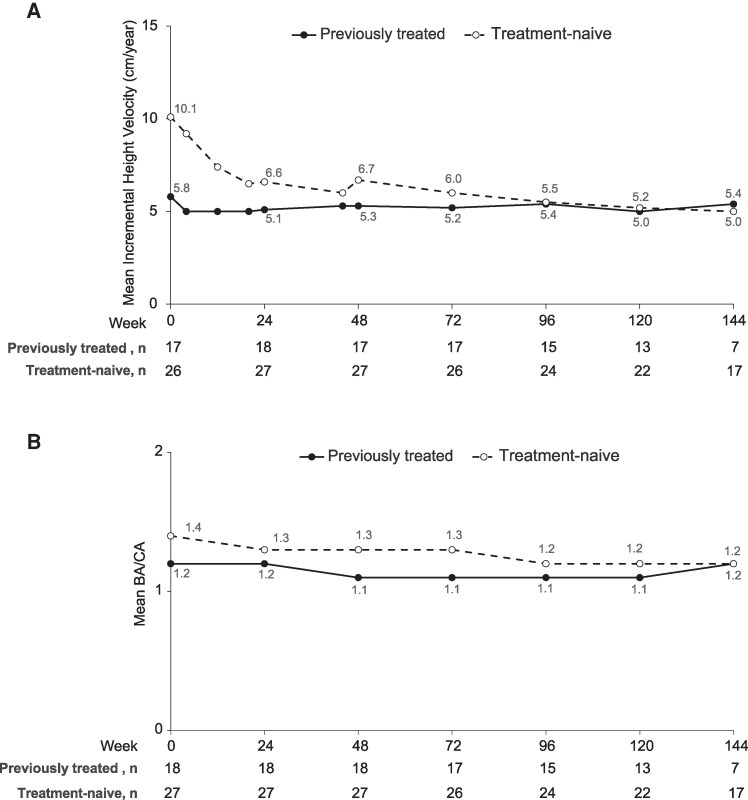
Mean (A) incremental height velocity and (B) BA/CA ratio in previously treated (solid) and treatment-naive (dotted) children. Week 0 indicates baseline assessment prior to first dose (ie, day 1 of treatment). Abbreviations: BA, bone age; CA, chronological age.

After an initial decline in treatment-naive children, the mean incremental height velocity (Fig. 6A) and BA/CA ratio (Fig. 6B) both remained relatively stable through week 144 and at levels expected with GnRHa treatment. Based on the available data from part 2, most children had a height velocity of ≥4 cm/year, 7 children had a height velocity between ≥3 and <4 cm/year, and 4 children had most of their height velocity measurements fall between ≥2.3 and <3 cm/year. A decline from baseline in BA/CA was observed in 95.3% to 97.4% of children between weeks 72 and 120, and in 100% at week 144. The mean BA/CA after 48 weeks of treatment was 1.2 in all children examined, indicating slower advancement in BA vs CA. The mean height SD score decreased from baseline or remained the same at all study visits after the third dose.

**Figure 7.**
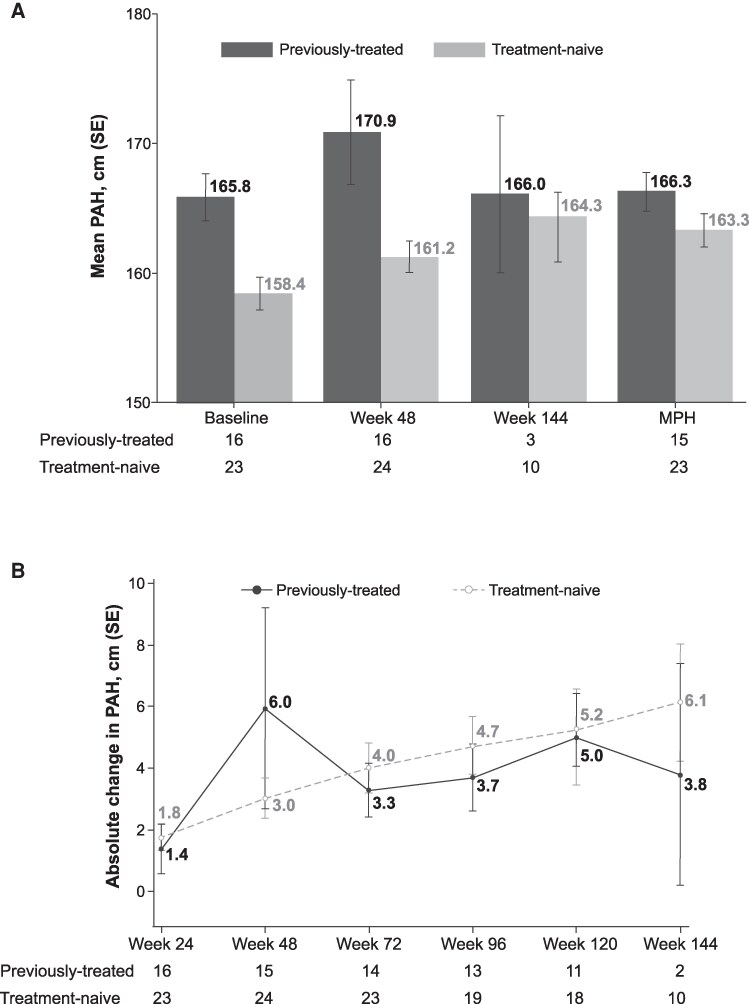
Mean (A) PAH and (B) absolute change in PAH in treatment-naïve or previously treated girls with available data. Abbreviations: MPH, mid-parental height; PAH, predicted adult height.

The greatest annualized change from baseline in PAH occurred by 48 weeks into treatment, at 4.9 cm/year (95% CI, −1.1, 10.9). After week 48, smaller changes were observed, and the annualized change from baseline in PAH at week 144 was 1.4 cm/year. In girls only, the mean PAH remained stable after week 48, and the mean PAH at week 144 was similar to mid-parental height (Fig. 7A). By week 144, treatment-naïve girls had gained 6.1 cm and previously treated girls 3.8 cm in absolute PAH over the duration of the study treatment period (Fig. 7B).

There was a slight decrease in BMI in previously treated children at week 144 (mean change in BMI [SD]: −0.2 [0.39]) and a slight increase in treatment-naive children (0.4 [0.73]), but there were no clinically meaningful changes from baseline in any children. Results were similar for all other time points analyzed.

### PROs

**Figure 8.**
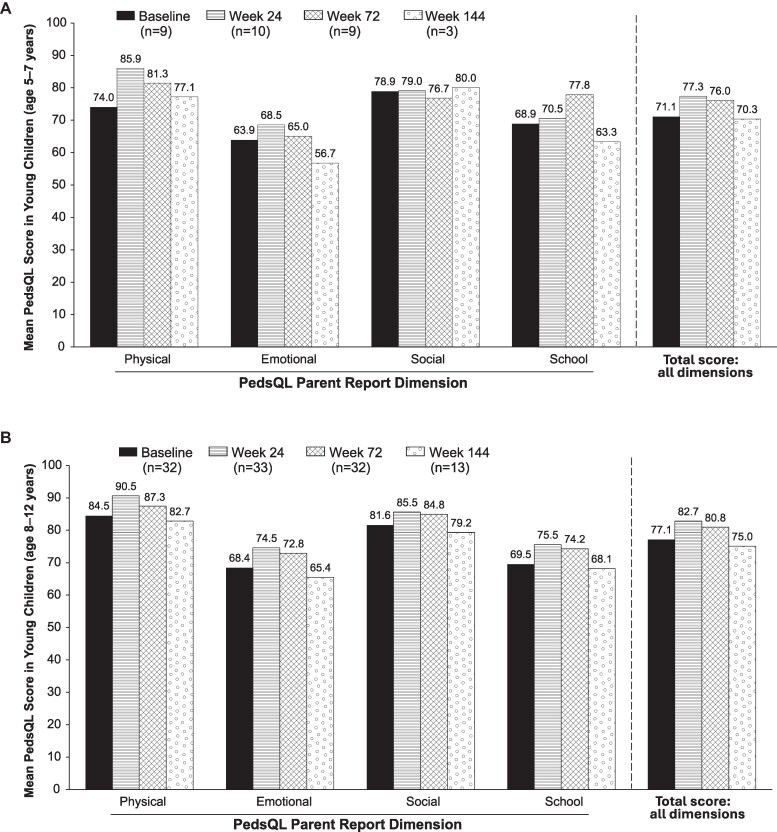
Pediatric Quality of Life Inventory^TM^ (PedsQL) parent-proxy reported dimension and total scores for (A) young children (5–7 years) and (B) children (8–12 years). Higher scores (from 0 to 100) indicate a better quality of life.

Owing to small sample sizes in different age groups, mean total scale PedsQL scores were plotted for children (8–12 years) and young children (5–7 years) but not for toddlers (2–4 years). Data were also combined for previously treated and treatment-naive children. Postbaseline mean total scores were similar to those observed at baseline among young children (Fig. 8A) and children (Fig. 8B). Slight increases were observed in total scale scores at weeks 24 and 72, with subsequent decreases at week 144 to approximately baseline scores. Similar change trajectories were observed for physical, emotional, and school functioning domain scores in young children and for all 4 dimensions of the PedsQL in children. However, the sample size declined among children from n = 32 to n = 13 from week 72 to week 144, with 3 young children examined at week 144. Toddlers showed slight numeric improvements from baseline to weeks 72 (n = 2) and 144 (n = 1) in all 4 dimensions of the PedsQL (physical functioning, 87.5–89.3 and 91.7; emotional functioning, 50.0–93.8 and 96.9; social functioning, 80.0–100 and 100; school functioning, 83.3–87.5 and 91.7).

**Figure 9.**
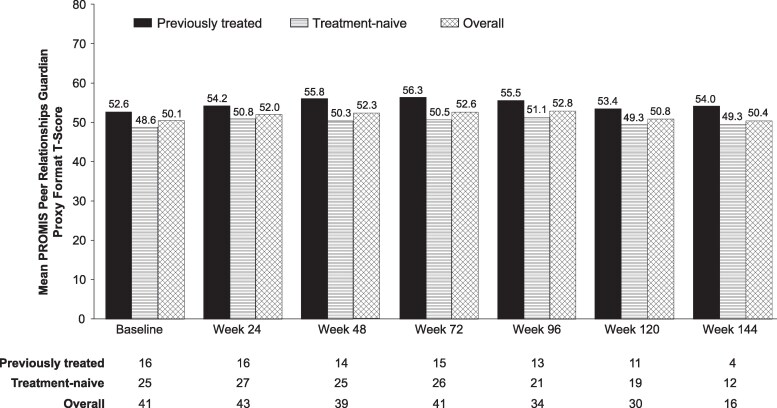
Mean Patient-Reported Outcomes Measurement Information System (PROMIS) peer relationships guardian proxy format T-scores in children ≥5 years of age. Analysis was based on nonmissing observations. Higher T-scores are indicative of better peer relationships; a score of 50 indicates the mean for a healthy population.

Mean PROMIS T-scores among children aged ≥5 years remained relatively similar after baseline through week 144 and close to or above the mean score of 50 for a healthy population (Fig. 9).

### Safety

All children reported an AE. The most common AEs were injection site pain occurring in 42 out of 45 children (93.3%) and other ISRs. Most AEs were mild or moderate in severity and were considered unrelated to the study drug by the investigator. Severe AEs were reported among 2 of 27 treatment-naive children in part 1 (1 child experienced severe events of disruptive mood dysregulation disorder and mood alteration, both considered related to LA 45 mg 6-month and both events resolved while the child continued treatment; 1 child experienced an intermittent severe event of trichotillomania, considered unrelated to study drug). In part 2, 1 previously treated child had a severe AE of tibia fracture, considered unrelated to LA 45 mg 6-month. No patients died or experienced AEs leading to study drug discontinuation. One serious AE (SAE) occurred throughout the entire study in part 1. A treatment-naive 8-year-old boy experienced an SAE of altered mood and a nonserious AE of disruptive dysregulation disorder on day 155; both events resolved on day 170 (before the second study drug dose) and were assessed by the site investigator as having a reasonable possibility of relationship to study drug. In terms of analysis of AESIs, 1 treatment-naive girl (aged 8 years) experienced a neuropsychiatric event of suicidal ideation on day 413 that resolved on day 840 and was assessed as having a reasonable possibility of being related to the study drug. She did not stop taking the study drug. None of the hypersensitivity events were related to the study drug. One mild AE of drug hypersensitivity was reported and was deemed to be related to pain medication. Bone fracture was uncommon during the study, and no subjects experienced events of convulsion or epiphysiolysis. No clinically meaningful laboratory or vital signs results were observed, and no new related safety concerns were identified.

## Discussion

This study reported up to just under 3 years of treatment safety and efficacy findings for LA 6-month formulation, which have not previously been reported [1, 2]. A comprehensive analysis of height-related outcomes, including PAH, was conducted, and the addition of PRO data contributes to a previous knowledge gap in the disease state. Treatment of CPP with LA 6-month IM depot over 144 weeks demonstrated sustained inhibition of the GnRH axis in children with CPP and maintenance in health-related quality of life. PAH increased with long-term treatment, further supporting long-term efficacy, while height velocity remained relatively stable and at prepubertal levels through week 144, and BA advanced slower than CA. Treatment was shown to be well tolerated throughout the study, and no new safety signals were identified.

PROs have been scarcely reported in clinical trials of GnRHa treatment in children with CPP [1, 3, 5, 18, 19]. This study provides valuable information based on a comparatively large sample size for this patient population and over 2.5 years of treatment. Children aged ≥5 years were found to have mean PROMIS peer relationships scores at week 144 that were at or above the mean for the US general population and similar to mean baseline scores. Overall, maintenance in health-related quality of life was observed based on the PedsQL Parent-Reported questionnaire in children (8–12 years), young children (5–7 years), and toddlers (2–4 years). In children and young children, small improvements from baseline in mean total PedsQL scale scores were initially seen over 72 weeks (≈1.5 years) of treatment, as well as in the underlying 4 domains, including the physical functioning component (except for social functioning in young children, which remained relatively stable). This was followed by mean decreases in the PedsQL scale scores from week 72 back to approximately mean baseline scores at 144 weeks (≈2.8 years) of treatment. However, sample sizes were substantially lower at week 144 vs week 72, and these results need to be interpreted with caution as it is unknown whether the results would have been different if the same population of children had been examined. It was unexpected to not have more improvement in PROs with treatment. The initial improvement is encouraging as children resume age-appropriate hormone levels and potential effects on psychosocial well-being are more consistent with those of their peers. Whether the lack of continuous improvement is related to the burden of ongoing medical treatment or the small sample size will need further study. Although our study did not demonstrate significant improvements in the PedsQL outcomes, the trend was positive with longer duration of treatment, and it is reassuring that no worsening was observed during long-term treatment.

In contrast to our findings, 2 prior studies have shown that GnRHa treatment may be associated with a decline from baseline in the physical functioning dimension of the PedsQL score [6, 7]. One prospective study observed a gradual and statistically significant decline from baseline in the physical functioning score after 1 and 2 years of GnRHa treatment; however, this was in a combined analysis of girls with CPP or with early-fast puberty (n = 32; age 7–10 years) [6 ]. Another cross-sectional study focusing only on children with CPP observed a statistically significant difference in the physical functioning score (77.54 ± 18.16 vs 86.43 ± 8.85) between 15 children with CPP currently being treated with a GnRHa (age 8–12 years) vs 30 age-matched controls without CPP [7]. However, the mean physical functioning score observed in children (age 8–12 years) in this prior study (77.54 ± 18.16) was generally lower than that observed in our study at baseline (84.5) or postbaseline through week 144 (range, 82.7–90.5). The reason for this trend is unclear. One possibility is that as GnRHa treatment suppresses sex hormone effects and thereby muscle development, physical functioning may decrease. In a cross-sectional survey study comparing children with CPP to healthy age-matched controls, mean PedsQL total scores were significantly lower in the CPP group (65.3 ± 1.8) compared to healthy controls (75.7 ± 1.2; *P* < .01) [20]. Similarly, both social and physical functioning scores were lower in CPP (62.4 ± 1.8 and 70.7 ± 2.2) vs healthy children (73.4 ± 1.2 and 79.9 ± 1.5, respectively). Across all subscales, scores for children with CPP ranged from 57.6 to 70.7, while scores for healthy children ranged from 69.8 to 79.9. Notably, in our study, PedsQL total scores for children and young children at all time points (baseline to week 144) were similar to the range reported for healthy controls in this cross-sectional survey and increased over time. Overall, further study is needed regarding the impact of GnRHa treatment on both physical functioning and the greater question of overall health-related quality of life.

In terms of height-related outcomes, mean height velocity remained relatively unchanged from a baseline mean of 5.8 cm/year through week 144 in previously treated children, and mean BA/CA ratio remained relatively unchanged. In treatment-naive children, mean height velocity remained stable after 48 weeks of treatment, and mean BA/CA declined by week 144. Overall, >95% of children experienced a decline from baseline in BA/CA at 72 to 144 weeks of treatment. PAH increased the most during the initial ≈12 months of treatment, with a maximum increase from a baseline of 4.9 cm observed at week 48 (95% CI, −1.1, 10.9). Beyond the initial 12 months of treatment, PAH remained stable, and the initial gains were maintained. The average gain in PAH by week 144 was 1.4 cm (95% CI, −1.4, 4.3). While a 1.4 cm/year increase in PAH may appear small, this reflects 5.2 cm of growth over 3 years and potentially more with longer duration of treatment and therefore still reflects a benefit in terms of maximizing final height in a population at risk for compromised adult stature. These observations are consistent with prior findings for IM LA depot among treatment-naive girls treated with 1- or 3-month formulations [21]. This study also reported the largest mean increase from baseline in annualized PAH by 12 months (4.8 cm). After this, smaller increases occurred, and the mean increase from baseline was 1.6 cm at 30 months (≈130 weeks) and 2.2 cm at 36 months (≈156 weeks) of treatment [21].

An excellent overall safety profile was observed with no new safety signals being identified. Mild or moderate AEs were primarily observed through week 144, of which injection site pain and other ISR were most common, and no patient experienced an AE leading to study drug discontinuation. One SAE occurred during the initial 48 weeks of treatment, which was deemed to be possibly related to the study drug. This SAE of altered mood resolved within 15 days, and the patient continued study treatment. One AESI of suicidal ideation was reported and was considered as potentially related to the study drug but was also transient and did not result in study drug discontinuation. The high incidence of children reporting ISR-related events in this study was likely due to the protocol-mandated requirement to capture as AEs all injection site reactions as a response in the Injection Site Assessment questionnaire completed after every study injection. All injection site reactions, whether associated with study drug administration or with the stimulation test, were required to be captured as AEs.

Strengths of this study include 3 years of treatment data, including boys, and examining PROs, which is not commonly assessed in clinical trials of GnRHa treatment of CPP [1, 3, 5, 18, 19]. Limitations include no active comparator (owing partly to CPP being a rare disease and challenges related to ensuring an adequate sample size), a smaller number of patients completing part 2 (as expected in part due to timing for treatment cessation), and a small number of boys being examined as expected (caution is needed when interpreting their data). Finally, although the sample size was relatively large for this patient population, analysis of PROs among different age groups did not allow for formal statistical comparisons of longitudinal changes. Future research could include longer-term follow-up, more detailed analyses of PROs data, and final height outcomes.

In conclusion, treatment of CPP with LA 6-month IM depot over 144 weeks demonstrated a sustained inhibition of the GnRH axis in children with CPP with an excellent safety profile. Maintenance in health-related quality of life was observed, and PAH improved over 3 years of treatment. PAH increased the most during the first year of treatment and continued to increase over 3 years.

## Data Availability

Some or all datasets generated during and/or analyzed during the current study are not publicly available but are available from the corresponding author on reasonable request. For more information on the process or to submit a request, visit the following link: https://www.abbvieclinicaltrials.com/hcp/data-sharing/.

## Clinical Trial Information

ClinicalTrials.gov identifier: NCT03695237 (registered October 2, 2018).
